# Postoperative Outcomes of Fontan Operation in a Multicenter Italian Study. How Far Have We Gone? Early Outcomes After Fontan Operation

**DOI:** 10.1007/s00246-024-03642-2

**Published:** 2024-09-17

**Authors:** Irene Cao, Emma Bergonzoni, Luca Vedovelli, Giulia Guerra, Lorenzo Galletti, Gianfranco Butera, Matteo Trezzi, Mario Panebianco, Gaetano D. Gargiulo, Emanuela Angeli, Lucio Careddu, Rossana Zanoni, Carlo Pace Napoleone, Luca De Orsola, Alvise Guariento, Fabio Scattolin, Alessandro Giamberti, Mauro Lo Rito, Stefano M. Marianeschi, Salvatore Agati, Ermanno Bellanti, Ugo Vairo, Giovanni Meliota, Gabriele Scalzo, Giuseppe Scrascia, Halkawt Nuri, Guido Michielon, Roberta Biffanti, Anna Gozzi, Giovanni Di Salvo, Vladimiro L. Vida, Massimo A. Padalino

**Affiliations:** 1 Department of Cardiac Surgery Unit, Thoracic, Vascular Sciences and Public Health, University of Padua, Padua, Italy; 2 Department of Cardiac Surgery, Cardiology and Heart and Lung Transplant, Pediatric Hospital Bambino Gesù, Rome, Italy; 3 Pediatric Cardiac Surgery and Adult Congenital Heart Disease Program, Department of Cardio-Thoracic and Vascular Medicine, IRCCS Azienda Ospedaliero-Universitaria Bologna, Bologna, Italy; 4 Pediatric and Congenital Cardiac Surgery, Regina Margherita Children’s Hospital, Turin, Italy; 5 Department of Pediatric and Adult Congenital Cardiac Surgery, IRCCS Policlinico San Donato, San Donato Milanese, Milan, Italy; 6 Pediatric Cardiac Surgery Unit, ASST Grande Ospedale Metropolitano Niguarda, Milan, Italy; 7 Department of Congenital Heart Surgery and Pediatric Cardiology Mediterranean Congenital Heart Center, “Bambino Gesù”-San Vincenzo Hospital, Taormina, Italy; 8 Department of Pediatric Cardiology, Giovanni XXIII Pediatric Hospital, Bari, Italy; 9 Department of Pediatric Sciences, Pediatric Cardiac Surgery Unit, Giovanni XXIII Pediatric Hospital, Bari, Italy; 10 Congenital Heart Surgery and Heart Centre, Gaslini Children’s Hospital, Genoa, Italy; 11 Division of Pediatric Cardiology, Departments of Women’s and Children’s Health, University of Padua, Padua, Italy; 12 Pediatric and Congenital Cardiac Surgery, Department of Precision and Regenerative Medicine and Jonian Area, University of Bari “Aldo Moro”, Bari, Italy

**Keywords:** Single ventricle, Fontan, Operative survival, Surgical palliation, Early outcomes, Univentricular heart

## Abstract

Despite the clinical results of the Fontan operation have certainly improved, it still presents with an inherent surgical risk of death and early morbidities. This is a retrospective clinical study of children undergoing Fontan operation in 9 congenital cardiac centers in Italy between 1990 and 2023. Clinical and surgical data were collected via a dedicated RedCap database. Primary outcome was cohort’s mortality, also considering different decades, while secondary outcomes were postoperative complications and reintervention. In the last 3 decades, there were 897 patients undergoing Fontan operation, M/F 512/384, median age: 4.5 years (IQR 3.3–6.4), median weight 16 kg (IQR 14–22). A first palliation was deemed necessary in 710 patients (80%), and most patients underwent a staged Fontan (93%); an extracardiac conduit was used in 790 patients (88%). Postoperative complications (mild to severe) occurred in 410 patients (46%), and early reinterventions were required in 66 patients (7.5%). Overall operative mortality was 1.7% (15 patients). Age at Fontan greater than 4 years was associated with an early need for transcatheter reintervention (adj *p* value = 0.037) and a higher incidence of postoperative complications (adj *p* value = 0.017). The Fontan operation has seen significant improvements in immediate outcomes, notably a remarkable reduction in overall mortality to just 1.35% in the last decade. While minor complications have remained steady, there has been a substantial decrease in major early complications, deaths, and the need for reinterventions. Notably, patients aged over 4 years seem to face a higher risk of postoperative morbidity, underscoring the critical role of age in preoperative assessment and management strategies for Fontan patients.

## Introduction

After the first repair for tricuspid atresia in Bordeaux in 1968 by Fontan and Baudet [1], surgical indications to the so-called Fontan palliation have progressively expanded to include various complex congenital heart diseases (CHD) with a functional single ventricle (FSV). Postoperative outcomes have shown a steady improvement over the years, and the high mortality rates experienced in the’80 s appear now to belong to the past [2–4]. Nevertheless, despite these achievements, the postoperative management after Fontan remains challenging, particularly because of the usually prolonged recovery period and the consistent incidence of pleural effusions. Additionally, the optimal age for Fontan completion continues to be a topic of debate [5].

We herein report our analysis in a large cohort of patients who underwent a Fontan completion in the last 3 decades, to assess current early results of Fontan operation and risk factors for early complications in a multicentric Italian study.

## Material and Methods

### Study Design

This is a multicenter retrospective observational study including patients with FSV undergoing Fontan completion between January 1990 and January 2023. This retrospective study was developed by the core institution (University of Padova, Italy). The study protocol conformed to the ethical guidelines of the 1975 Declaration of Helsinki, as reflected in approval by each participating site’s ethical regulatory body. The overall study was approved by the Institutional Review Board at the University of Padova (nr. 20,616, CESC code: 5423/AO/22) and by each participating center. The requirement for informed consent was waived given the study design.

Clinical and surgical data were retrieved from medical records by local investigators and recorded in a secure online RedCap database between December 2021 and January 2023.

Primary outcomes were early mortality (during hospitalization or within 30 days after surgery) and the analysis of the variation of its incidence across the last three decades. Secondary outcomes were early reinterventions, both catheter and surgical, and postoperative complications.

Requested preoperative and postoperative data are described in detail in Tables 1, 2, and 3.

**Table 1:** Groups including all cardiac diagnosis according to anatomical similarities and the presence of a significant additional chamber

Group 1	Complex type	transposition of the great arteries, double outlet right and left ventricle, mitral atresia, criss cross heart, Ebstein anomaly
Group 2	Right ventricular hypoplasia	tricuspid atresia, pulmonary atresia with intact ventricular septum
Group 3	Left ventricular hypoplasia	hypoplastic left heart syndrome and hypoplastic left heart complex
Group 4	Double inlet type	double inlet right ventricle and double inlet left ventricle
Group 5	Unbalanced atrioventricular septal defect type	unbalanced atrioventricular septal defect

**Table 2:** Patients’ characteristics and surgical operative data

Variables	All
Patients	897
Male	512 (57%)
Age at Fontan	4.5 (IQR 3.3–6.4)
Weight at Fontan	16 (IQR 14–22)
Prenatal Diagnosis	207 (58%)
Unknown	540
Secondary/Additional Ventricular Chamber	199 (23%)
Heterotaxy Syndrome	80 (8.9%)
Syndrome/Chromosomal Abnormalities	22 (2.5%)
Main cardiac diagnosis	
Right ventricular hypoplasia	283 (32%)
Complex type	256 (29%)
Left ventricular hypoplasia	174 (19%)
Double inlet type	122 (14%)
Unbalanced atrioventricular septal defect type	62 (6.9%)
Dominant Left /Right ventricle	479 (53%)/370 (41%)
Undefined	48 (5.4%)
First palliation	710 (80%)
Systemic to pulmonary artery shunt	222 (25%)
Norwood type, Damus-Kaye-Stensel	224 (25%)
Pulmonary artery band	148 (17%)
Other	116 (13%)
Staged Fontan	829 (93%)
Bidirectional Glenn	748 (84%)
Bilateral Glenn	37 (4.2%)
Kawashima	18 (2%)
Hemifontan	13 (1.5%)
Other	13 (1.5%)
CPBP during Fontan staging	470 (96%)
Fontan Type	
TCPC, Extracardiac ((median conduit 18 (IQ 16,20)	790 (88%)
TCPC, Lateral Tunnel	87 (9.7%)
Kreutzer, Bjork	12 (1.3%)
Hepatic vein to Azygous Baffle, unknown, other	5 (0.6%)
Fenestration	467 (52%)
CPBP at Fontan completion	487 (95%)

**Table 3:** Main postoperative data after Fontan Operation

Postoperative characteristics	All patients
Onset of postoperative complication	410 (46%)
Pleural effusion requiring chest tubes stay > 3 days	283 (32%)
Pleural effusion requiring chest tubes stay > 7 days (out of 269 patients)	241 (85%)
Unknown	370
Infection requiring antibiotic therapy > 7 days	63 (7%)
Chylothorax	36 (4%)
Bradicardia	33 (3.7%)
IPPV > 24 h	25 (2.8%)
Fontan takedown	20 (2.2%)
Tachycardia	20 (2.2%)
AKI	19 (2.1%)
LCO syndrome	17 (1.9%)
Any reoperation	17 (1.9%)
Bleeding requiring reoperation	16 (1.8%)
Respiratory failure requiring IPPV > 3 days	15 (1.7%)
Any cerebrovascular/neurological with complete resolution	9 (1%)
Hemi-diaphragm paralysis	8 (0.9%)
Any cerebrovascular/neurological with residual deficit	6 (0.7%)
hemoptysis pulmonary bleeding	2 (0.2%)
Other	49 (5%)
ICU stay (days), median, IQR)	3 (2, 4)
Early Mortality	15 (1.7%)
Early Reintervention	66 (7.5%)
Yes, surgical	51 (5.8%)
Yes, catheter intervention	15 (1.7%)

Perioperative data included purely demographic and anatomical information (gender, prenatal diagnosis, type of congenital heart disease, ventricular dominance, the presence of an additional ventricular chamber as described elsewhere) [6]. As mentioned, heterotaxy syndromes, extracardiac abnormalities, chromosomal abnormalities, Fontan intervention date and type (including procedure type—Bjork, Kreutzer, extracardiac conduit-ECC, lateral tunnel-LT—fenestration, palliation, and staging), use of extracorporeal circulation, need for deep hypothermia, complications, early mortality, any reinterventions, therapy, and discharge date were collected. In terms of postoperative complications, prolonged pleural effusions, arrhythmias, infections requiring antibiotic therapy for more than 7 days, chylothorax, transitional neurological events, and hemi-diaphragmatic paralysis were classified as minor adverse events (AEs), given their resolution without any clinical deficit. Major AEs included postoperative low cardiac output syndrome (LCOS), Fontan takedown, the need for prolonged mechanical support, major neurological events resulting in residual clinical disability, respiratory failure requiring invasive positive pressure ventilation (IPPV) for more than 3 days, acute kidney injury (AKI), bleeding necessitating reoperation, early death (within 30 days or during the hospital stay), or the need for any reintervention (surgical or percutaneous). Additionally, the study population was divided into three decades to gain a better understanding of advancements in the management of Fontan patients and to highlight differences in both surgical procedures and outcomes achieved. Last, the main cardiac diagnoses were arbitrarily divided into 5 groups (Table 1), according to the anatomical similarities and the presence of significantly large additional ventricular chambers, as above mentioned.

### Statistical Analysis

Continuous variables were described as medians (interquartile range [IQR]) and categorical variables as percentages. Statistical analysis was conducted using Fisher exact tests for categorical variables and non-parametric Mann–Whitney U tests for continuous variables. P values were corrected for false discovery rate using the Benjamini–Hochberg method. Regressions were carried out as a generalized linear mixed model (GLMM) to accommodate both fixed (e.g., age at Fontan and cardiac diagnoses) and random effects. Specifically, the variable “center” identifying each institution was included as a random effect to account for the intragroup correlation inherent to the multicentric design of the study. All statistical analyses were carried out in the R (v.4.3.2) statistical computing environment.

## Results

This study includes 893 patients (Male/Female = 512/384) undergoing Fontan operation in 9 Italian tertiary centers for congenital cardiac disease from January 1, 1990, to January 31, 2023. Median age and weight at Fontan were 4.5 years (IQR 3.3–6.4) and 16 kg (IQR 14–22), respectively. Prenatal diagnosis was available in 207 patients (58%).

Dominant ventricular morphology was left in 479 patients (53%), and right in 370 patients (41%). An additional ventricular chamber was reported in 199 patients (23%). Heterotaxy syndrome was diagnosed in 80 patients (8.9%).

The main cardiac diagnoses (Table 1, 2) were Right ventricular hypoplasia in 283 patients (32%), complex type in 256 patients (29%), Left ventricular hypoplasia in 174 patients (19%), Double inlet type in 122 patients (14%), and unbalanced atrioventricular septal defect type in 62 patients (6.9%). Only 22 patients were associated with chromosomal abnormalities or syndromes (2.5%).

**Fig. 1:**
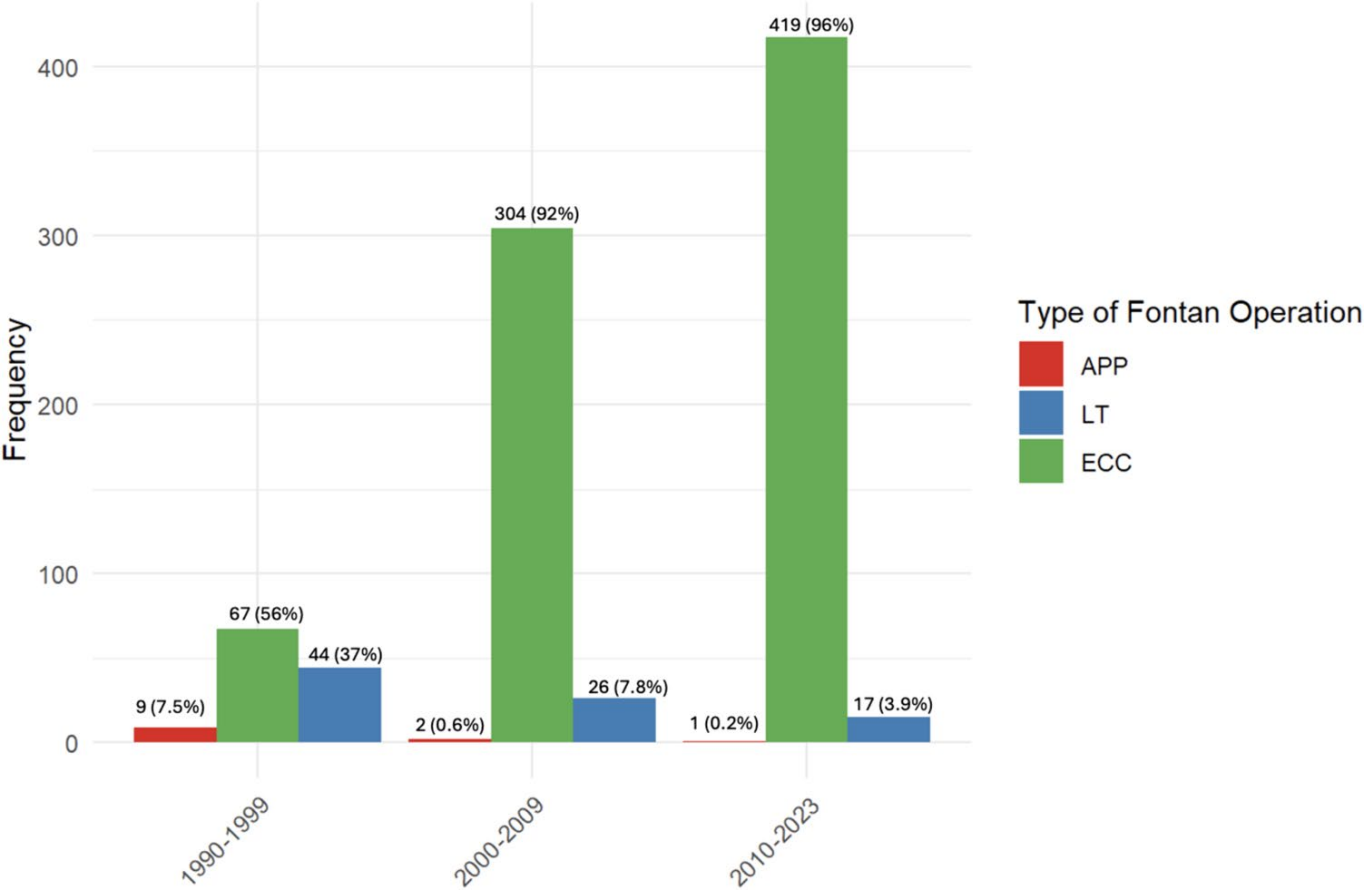
Type of Fontan Operation through the last three decades

An initial palliation procedure was conducted in 710 patients (80%), and the majority of patients underwent a staged Fontan pathway (829 patients, 93%), involving cavo-pulmonary anastomosis in 785 patients (88.2%). Fontan completion was predominantly achieved using an ECC in 790 patients (88%) (median conduit 18 (IQ 16, 20), followed by the LT technique in 87 patients (9.7%), and atrio-pulmonary (Kreutzer) or atrial-ventricular (Bjork) connection in 12 patients (1.3%). Other or unspecified techniques were employed in 5 patients (0.6%) (Table 2). A fenestration was performed in 467 patients (52%) (Table 2). Notably, the utilization of ECC exhibited a progressive increase and emerged as the most prevalent surgical technique in recent years (Fig. 1).

The overall incidence of complications in the entire population was 46% (410 patients) (Table 3). Given the most common Fontan complication, namely pleural effusions, we lacked data on the exact drainage maintenance (days) for 370 patients (41.4%). However, among all the remaining, 283 had chest tubes left in place for more than 3 days, as anticipated due to the substantial susceptibility of Fontan patients to effusions. Additionally, among these patients, the median duration of chest tube placement was 7 days, with 241 out of 283 (85%) requiring pleural drainage for more than 7 days.

Early reinterventions (within 30 days or during the same hospitalization) were required in 7.5% (66 patients): surgical in 51 (5.8%) and interventional-percutaneous in 15 (1.87%). Early mortality was 1.7% (15 patients). Main postoperative data are summarized in Table 3.

**Fig. 2:**
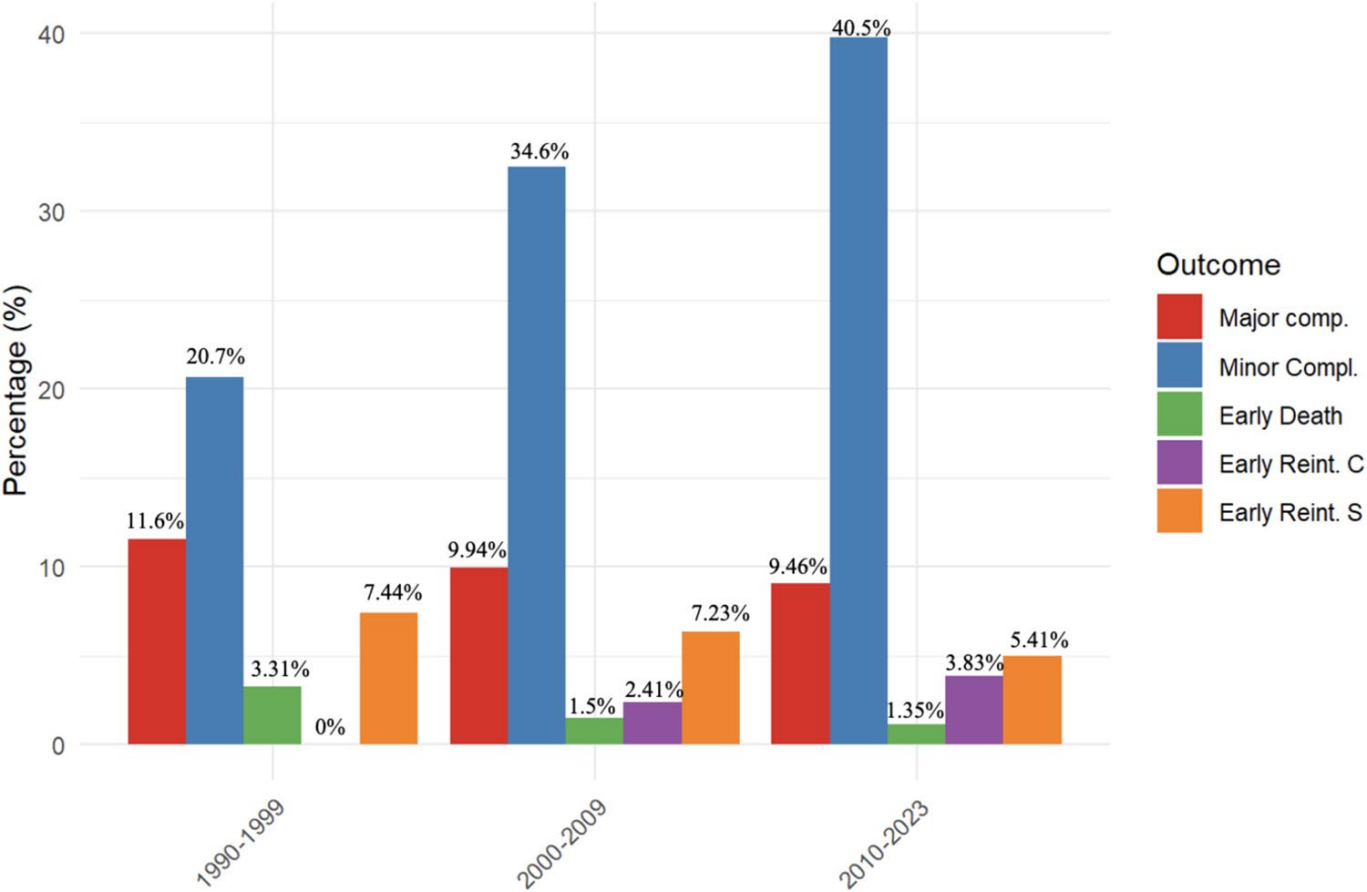
Representation of the incidences of the main postoperative complications divided by decade. C = catheter; S = surgical

Notably, the total number of patients undergoing Fontan operation has increased consistently over time, while early mortality and incidence of surgical reinterventions have drastically decreased (from 3.31% to 1.35%, and 7.44% to 5.41%, respectively) (Fig. 2,).

There were no statistically significant differences in terms of postoperative complications, mortality, and rate of early reinterventions between genders (*p* value 0.9), nor according to the main cardiac diagnosis (*p* value 0.4, 0.5, 0.5 respectively), dominant ventricular morphology (*p* value 0.7, 0.8, 0.4 respectively), or the presence of an additional ventricular chamber (*p* value > 0.9, 0.8, > 0.9 respectively).

It is of note that the onset of postoperative chylothorax was significantly associated with CHD with right ventricular dominance (FSRV) when compared to left ventricle dominance (*p* value 0.03). Additionally, the presence of a surgical fenestration was associated with a higher incidence of percutaneous reinterventions (*p* value 0.015), while non-fenestrated patients exhibited a greater tendency toward drainage duration exceeding 3 days (*p* value 0.002) (Table 4).

**Table 4:** Analysis of the associations between some demographic’s features and the incidence in the main postoperative outcomes

	Fenestration: No Yes	Age at Fontan: > 4 years < 4 years
Early death	7 (1.6%) 8 (1.7%)	6 (1.1%) 9 (2.5%)
Adj *p* value	0.9	0.2
Postoperative Complication	196 (46%) 214 (47%)	265 (50%) 145 (40%)
Adj *p* value	0.9	0.017
LCO syndrome	4 (0.9%) 13 (2.8%)	9 (1.7%) 8 (2.2%)
Adj *p* value	0.1	0.8
Arrhythmias (Bradi)	15 (3.5%) 18 (3.9%)	19 (3.6%) 14 (3.9%)
Adj p value	0.9	0.9
Pleural effusion > 3 days	199 (88%) 232 (77%)	312 (86%) 119 (72%)
Adj p value	0.019	< 0.001
Early reintervention surgical	27 (6.3%) 30 (6.4%)	28 (5.2%) 29 (8%)
Adj p value	0.9	0.2
Early reintervention catheter	6 (1.4%) 19 (4.1%)	21 (3.9%) 4 (1.1%)
Adj p value	0.015	0.037
Early reintervention surgical + catheter	33 (7.7%) 45 (9.6%)	45 (8.4%) 33 (9.1%)
Adj *p* value	0.6	0.9

When comparing ECC vs LT, in this series, the latter was associated with a greater incidence of LCO syndrome and early mortality (adj *p* value 0.024 adj *p* value 0.005) (Table 5).

**Table 5:** Analysis of the associations between type of Fontan Operation and the incidence in the main postoperative outcomes

Characteristics	Kreutzer/Bjork (*N* = 12)	Hepatic vein to Azygos baffle (*N* = 5)	TCPC extracardiac conduit (*N* = 788)	TCPC Lateral tunnel (*N* = 85)	Kreutzer/Bjork vs Hepatic vein to azygos baffle adj *p*-value	Kreutzer/Bjork vs TCPC extracardiac conduit adj *p*-value	Hepatic vein to azygos baffle vs TCPC extracardiac conduit adj *p*-value	Kreutzer/Bjork vs TCPC lateral tunnel adj *p*-value	Hepatic vein to azygos baffle vs TCPC lateral tunnel adj *p*-value	TCPC extracardiac conduit vs TCPC lateral tunnel adj *p*-value
Postoperative complications	9 (75%)	5 (100%)	345 (44%)	48 (57%)	0.3	0.062	0.06	0.3	0.091	0.06
LCO syndrome	1 (8.3%)	0 (0%)	11 (1.4%)	5 (5.7%)	0.8	0.15	0.8	0.8	0.8	0.024
Early death	0 (0%)	0 (0%)	9 (1.1%)	5 (5.8%)	-	0.8	0.8	0.8	0.8	0.005
Early surgical reintervention	0 (0%)	1 (20%)	52 (6.6%)	4 (4.6%)	0.5	0.5	0.5	0.5	0.5	0.5
Early catheter reintervention	0 (0%)	0 (0%)	22 (2.8%)	1 (1.1%)	-	0.8	0.8	0.8	0.8	0.8
Pleural effusion > 3 days	6 (75%)	4 (80%)	373 (86%)	46 (61%)	> 0.9	0.6	0.8	0.6	0.6	< 0.001

Of note, we observed that age exceeding 4 years at Fontan completion was significantly correlated with a higher incidence of postoperative complications (*p* = 0.017), particularly concerning the requirement for early non-surgical reintervention (*p* = 0.037) and higher incidence of pleural drainage maintenance (*p* < 0.001) (Table 5). In conducting logistic regression analysis, with age treated as a continuous variable and adjusted for fenestration, age emerged as an independent risk factor for the composite outcome of postoperative complications, early reintervention, and early mortality. Specifically, with each additional year of age at the time of the Fontan procedure, the risk of experiencing all three outcomes increased by 7% (Table 6).

**Table 6:** Logistic regression analysis with occurrence of postoperative complications as dependent variable and age at Fontan and fenestration as covariates

Characteristic	OR	95% CI	*p*-value
Age at Fontan	1.07	1.03–1.1	< 0.001
Fenestration			
no	-	-	
yes	1.08	0.83–1.41	0.6

However, in regression analysis, when age was further adjusted for other variables (main cardiac diagnosis or type of Fontan surgery), the impact of age disappeared when considering anatomical and surgical factors (*p* value 0.8, 0.9, respectively). This suggests that the tendency toward increased complications is more closely associated with underlying anatomical conditions rather than age at surgery.

**Fig. 3:**
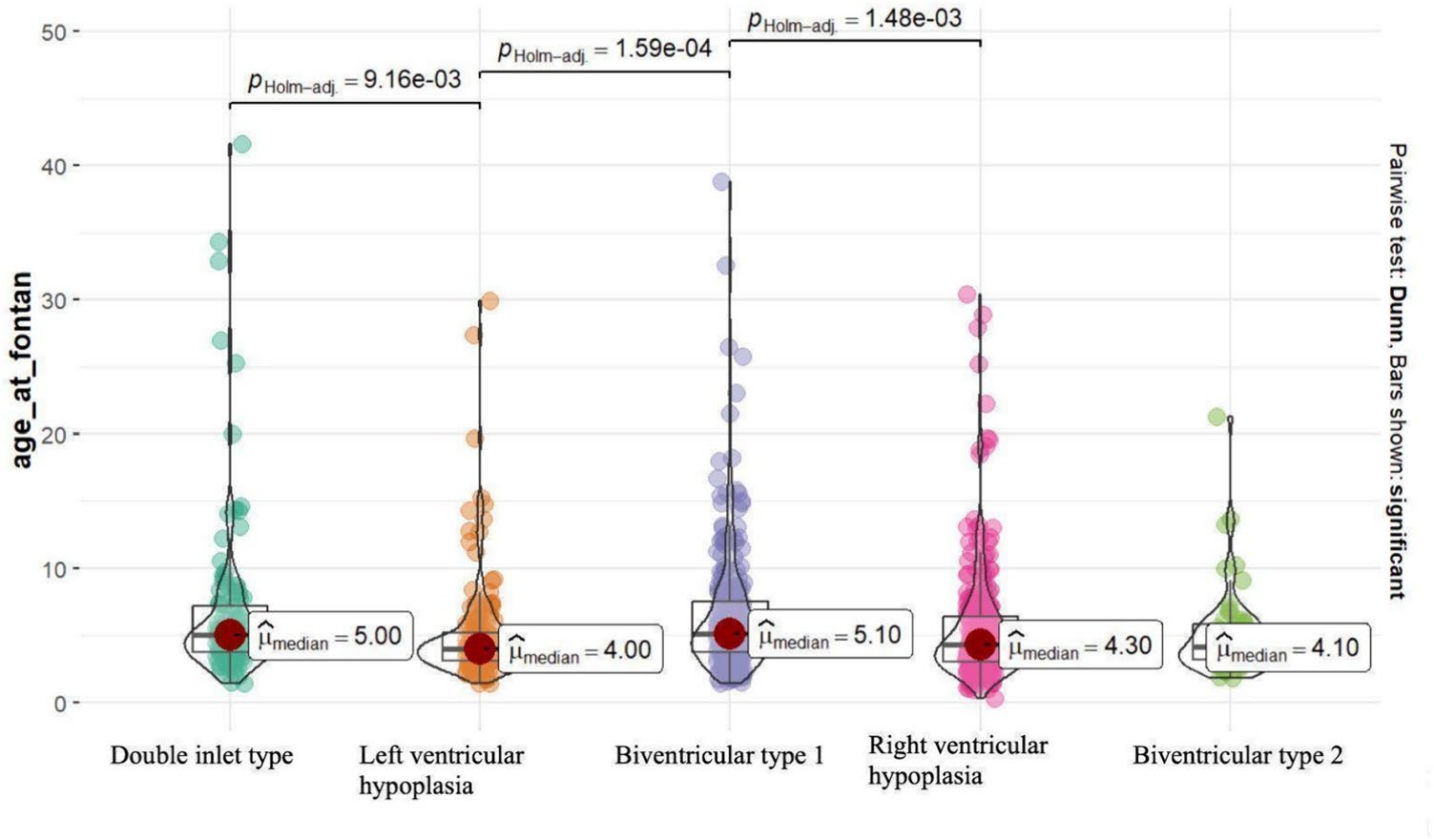
Boxplot showing the distribution of age at Fontan concerning the type of cardiac diagnosis. μ median = median age

Regarding cardiac diagnosis, the median age at Fontan for each group of heart defects was calculated, revealing a statistically significant difference between the groups (adjusted *p* value < 0.001). A boxplot illustrating the distribution of age at Fontan relative to the type of cardiac diagnosis is presented (Fig. 3).

Last, median age of patients undergoing ECC was significantly higher when compared to LT (4.6 years-IQ 3.4–6.5, versus 3.5-IQ 2.5–5, adjusted *p* value < 0.001).

## Discussion

Fontan palliation has emerged as a standard procedure for patients with FSV. Since its inception [1], there have been significant improvements in the early outcomes of Fontan circulation. The mortality rate, once high in the 1980s [7, 8], has decreased to approximately 2–4%, according to data from the ECHSA congenital database (https://echsacongenitaldb.org) and other previously cited reports [2–4]. Over the past thirty years, the original Fontan surgical technique has undergone various modifications, evolving from the initial techniques proposed by Bjork and Kreutzer [9, 10] to the refined methodology introduced by Marcelletti [11]. In our study population, the ECC has emerged as the preferred surgical approach (Fig. 1), followed by the LT [12–14], particularly in younger pediatric patients, as it avoids the use of small conduits which may cause a need for a late reoperation for conduit replacement.

It appears that despite the use of diverse surgical techniques (namely ECC vs LT), there was not a significant difference in the occurrence of early complications overall. However, it is noteworthy that LT has shown a stronger association with the onset of LCO syndrome and early mortality compared to ECC. This difference in outcomes may be influenced by various factors (such as the patients’ BSA and age at operation, need to operate earlier). Additionally, in other studies comparing the LT vs ECC procedures, the former has been linked to higher rates of postoperative complications, particularly arrhythmias. This suggests that the choice of surgical approach may have implications for patient outcomes and warrants careful consideration in clinical decision-making [15–19].

Within our study population, there has been a remarkable and continuous reduction in mortality, with an overall mortality rate of 1.7%, and an evident dropping from 3.31% in the 1990s to 1.35% more recently (Fig. 2). This trend is further highlighted when examining the data by decades, revealing a consistent decrease in mortality rates over time. This decline can be attributed to advancements in surgical techniques, improvements in preoperative patient selection, and enhancements in postoperative management protocols. Conversely, there has been a notable increase in overall complications, impacting a substantial portion of our cohort (46%). This trend aligns with previous findings reported in 2012, where a large multicenter study involving 2747 patients undergoing TCPC with lateral tunnel (LT) versus extracardiac conduit (ECC) in the USA documented a complication rate of 40.4% [20, 21].

However, upon scrutinizing these complications, it becomes evident that minor complications and the need for non-surgical reintervention have escalated (from 20.7 to 40.5%, from 0 to 3.83% respectively), juxtaposed with a reduction in total major complications (from 11.6 to 9.46%) and surgical need of reintervention (from 7.44 to 5.41%) (Fig. 2). This trend through decades in mortality and incidence of complications mirrors the one observed in other scientific studies. In an Italian report published in the 90 s, the rate of early mortality was 10%, of early Fontan failure was 15%, of Fontan takedown was 10%, and of overall surgical reintervention was 16.6% (Fontan takedown, stenotic anastomosis, plication of hemidiaphragm) [22]. In contrast, in a more recent and previously cited study, released in 2012, the researchers analyzed 2747 Fontan patients and the in-hospital mortality turned out to be 1.6% with a Fontan takedown/revision rate of 1.4% [20].

Persistent pleural effusions and prolonged chest tube stay are well-known postoperative complications after Fontan [1, 3, 4, 7, 15, 19–21]. In our series, the median duration of chest tube placement was 7 days, consistent with findings reported by others [15]. Also, 241 patients (89.6% of those requiring prolonged chest tube stay) had their chest tubes in place for more than 7 days. This extended timeframe confirms the persistent nature of the postoperative effusion issue, potentially influenced also by the prevalent practice of prolonged chest tube insertion in Fontan patients, who have a known propensity for effusion. Notably, a higher percentage of non-fenestrated patients had prolonged chest tube retention compared to fenestrated patients (88 vs. 77%; *p* value 0.019). This finding underscores the potential impact of fenestration on postoperative drainage management in Fontan patients. Further data analyses are necessary to delve deeper into this observation and understand its implications fully.

Notably, when assessing the clinical impact of ventricular dominance on early outcomes, we did not find any statistically significant differences in the occurrence of major adverse events, other than a higher incidence of chylothorax in patients with right ventricular dominance (5.7 vs 2.5%; *p* value 0.03). Recent findings by Ponzoni et al. suggested that Fontan patients with a right-dominant ventricle exhibit inferior long-term survival, especially when the anatomical right ventricle is part of the systemic circulation [23]. Also, Pollak et al. [24] highlighted prolonged postoperative hospitalization, along with poorer early postoperative indicators (ventricular dysfunction and atrioventricular valve regurgitation), in patients with right ventricular morphology compared to those with left ventricular morphology. A study by Ovroutski et al. [25] suggested right ventricle morphology as a risk factor for early Fontan failure. Our observations suggest that the early postoperative course following the Fontan operation may be influenced by the effective systolic and diastolic function of the ventricular chamber, regardless of its morphology.

One noteworthy finding from our research pertains to the age at Fontan completion. Presently, there exists no consensus among clinicians or surgeons regarding the optimal timing of Fontan completion. Some studies have reported no statistically significant differences in early outcomes, morbidity, or mortality based on age [26, 27]. Other reports showed that an age of approximately 4 years or younger, at the time of Fontan completion, is associated with better outcomes, both in the short and long term [28]. In contrast, higher age has been associated with the need for revision surgery, and late death [19, 29], Fontan failure, a decline in exercise capacity, and a reduction in cardiac index [30–33]. In a study of 3319 patients, published by Akintoye et al. in 2018, the age of 3 years was identified as the optimum for Fontan completion, as evidenced by a lower rate of in‐hospital mortality, procedure‐related complications, and rate of nonroutine home discharge [28]. However, a study by Pace Napoleone et al. [34] affirmed that the modified Fontan operation can be performed safely in older patients without affecting operative and medium-term follow-up results. They divided their cohort into two groups according to the age of 7 years old at the time of Fontan completion. Mortality (respectively 0% in group < 7 years, 5.4% in group > 7 years; *p* = 0.5) and complications were similar between the two groups. As D’Udekem et al. reported [35], the ‘ticking clock theory’ advocates that any Fontan circulation will have a limited lifetime so the decision regarding the age at which to perform the Fontan procedure is challenging. Delaying the intervention poses a high risk of paradoxical embolism and side effects associated with chronic cyanosis. On the other hand, performing the procedure early entails a heightened risk of elevated pulmonary resistance, systemic venous hypertension, arrhythmias, and low oxygen saturation. In our cohort of Fontan patients, the median age was 4 years, which we used as a cutoff to divide our cohort. Upon analyzing the overall incidence of complications, we observed that patients older than 4 years exhibited a higher occurrence of poor early outcomes compared to those younger than 4 years (50 vs 40%; adjusted *p* value 0.017). We also noted a correlation with percutaneous reinterventions (3.9 vs 1.1%; adjusted *p* value 0.037) and drainage duration exceeding 3 days (86 vs 72%; adjusted *p* value < 0.001). Our logistic regression analysis, which included age as a continuous variable and was adjusted for fenestration, identified age as an independent relative risk factor. Specifically, for each additional year at the time of Fontan operation, there was a 7% increase in the risk of encountering complications. However, upon additional adjustment for other variables such as main cardiac diagnosis or type of Fontan operation, the significance of age as a risk factor diminished. This suggests that despite younger age seems to be a protective factor for early outcomes, the impact of age on complication risk may be also influenced by the specific procedural approach utilized and the underlying cardiac pathology, rather than age itself being an inherent risk factor.

## Study Limitations

As a retrospective multicenter study, there may have been selection bias; however, we endeavored to mitigate this bias by including centers with varying Fontan completion volumes and encompassing all Fontan procedures at each center. While we have comprehensive preoperative and operative data on all enrolled patients, disparities in postoperative ICU time and missing data could have impacted our results. Additionally, due to the extensive time frame covered by our cohort, the loss of certain anatomical details and perioperative data was unavoidable and may have influenced our findings.

## Conclusion

The perioperative outcomes of the Fontan procedure have shown a consistent and notable improvement over the past thirty years in Italy. Mortality rates have steadily decreased from 3.31 to 1.35% across the decades. Among the various surgical techniques utilized, the ECC method has emerged as both the most frequently employed and the safest option, associated with fewer postoperative complications compared to alternative approaches. However, our analysis has revealed that age exceeding 4 years at the time of Fontan completion may represent a risk factor for the development of complications, reinterventions, and early mortality, not merely due to age itself, but because of the factors typically associated with this age group. This emphasizes the critical role of considering patient age as a pivotal factor in the planning and execution of the Fontan procedure. By carefully considering age in surgical decision-making, healthcare professionals can strive for optimal outcomes and mitigate potential risks associated with Fontan surgery.

## Data Availability

Data that support the findings of this study have been collected and deposited in https://research.dctv.unipd.it/redcap/ Data are protected with user and password and avaiable through reasonable request.
